# Black tea-processed *Toona sinensis* leaves alleviate DSS-induced ulcerative colitis in mice by enriching flavonoids and modulating gut microbiota

**DOI:** 10.3389/fnut.2026.1888003

**Published:** 2026-07-14

**Authors:** Mengdi Wang, Shuolei Xu, Jinyi Liu, Ruojue Xu, Boyu Du, Guangshun Lu, Guohuo Wu

**Affiliations:** 1 Department of General Surgery, The First Affiliated Hospital of Fuyang Normal University, Fuyang, China; 2 School of Biology and Food Engineering, Fuyang Normal University, Fuyang, China; 3 The First Clinical Medical College, Bengbu Medical University, Bengbu, China

**Keywords:** anti-ulcerative colitis, black tea processing, flavonoid, gut microbiota, intestinal barrier, phytochemicals, Toona sinensis leaves, ulcerative colitis

## Abstract

Ulcerative colitis (UC) is a chronic inflammatory bowel disease for which safe dietary interventions remain limited. *Toona sinensis* leaves, a traditional medicinal food rich in polyphenols and flavonoids, exhibit anti-inflammatory potential; however, how processing techniques modulate their functional nutrition and anti-colitis activity is poorly understood. In the present study, we compared hot air-dried *T. sinensis* leaves (TL) and black tea-processed leaf tea (TLT) in a mouse model of DSS-induced colitis (*n* = 8 per group) at a dose of 20 mg/ml (approximately 3.33 g/kg body weight per day) via drinking water, with Keemun black tea (BT) as a positive plant-based dietary control. Both TL and TLT significantly mitigated DSS-induced colitis; notably, TLT showed significantly stronger efficacy than TL (*p* < 0.05) and exhibited efficacy comparable to BT. TLT restored colonic histopathology, upregulated tight junction proteins (ZO-1 and Occludin), and suppressed pro-inflammatory cytokines (IL-1β and TNF-α). Black tea processing of *T. sinensis* leaves increased bioactive components including total flavonoids and polyphenols, which strongly correlated with protective indicators (|r| > 0.6, *p* < 0.01). TLT also restored gut microbial diversity and enriched SCFA-producing genera such as *Akkermansia, Odoribacter*. Untargeted metabolomics identified 23 key anti-inflammatory metabolites including kaempferol and quercetin glycosides. Multi-omics integration revealed significant correlations among metabolites, gut microbiota, and protective endpoints. Collectively, these results demonstrate that dietary intervention with black tea-processed *T. sinensis* leaves alleviates UC primarily by increasing flavonoid content, protecting intestinal barrier integrity, and remodeling gut microbiota homeostasis. This study provides a theoretical basis for the value-added utilization of *T. sinensis* leaves as a functional food for dietary intervention against inflammatory bowel disease.

## 1 Introduction

Ulcerative colitis (UC) is a chronic relapsing inflammatory bowel disease (IBD) characterized by mucosal erosion, epithelial barrier disruption, inflammatory infiltration, and gut microbiota dysbiosis, with a steadily rising global prevalence (1, 2). UC pathogenesis is multifactorial, involving genetic susceptibility, environmental triggers, gut dysbiosis, and aberrant immunity. Key features include Th1/Th17 activation, overproduction of TNF-α and IL-1β, and impaired intestinal barrier function (3). Major risk factors comprise a Western-style diet, early-life antibiotic use, and family history of IBD (4). UC predominantly affects young adults (20–40 years), with a slight male predominance. Global prevalence is highest in North America and Europe (~0.3–0.5%), while Asian countries, including China, have seen a rapid rise over the past two decades, indicating an emerging public health concern (5, 6). Current treatment options, including 5-aminosalicylates, glucocorticoids, and biologics, are limited by side effects, high costs, and incomplete mucosal healing (7). Therefore, natural bioactive compounds from foods have attracted growing interest as safe and accessible complementary approaches for UC management.

*Toona sinensis* (Chinese toon) is a deciduous tree native to East Asia and widely cultivated in China, Japan, Korea, and Southeast Asia. In China, major production areas include Anhui, Henan, and Sichuan provinces. Its tender young leaves, harvested in early spring, are traditionally consumed as a seasonal vegetable prized for their distinctive aroma and flavor (8, 9). Mature leaves are often dried and brewed as a functional tea, especially in rural central and southern China. *T. sinensis* leaves are affordable, accessible, and nutritionally rich, containing dietary fiber, protein, vitamins C and E, and essential amino acids (10). Economically, it represents an underutilized forest product with growing market potential in the functional food industry. Phytochemical studies have revealed that *T. sinensis* leaves are rich in flavonoids, polyphenols, terpenoids, and vitamins, which confer anti-inflammatory, antioxidant, and intestinal protective properties (10). However, fresh leaves are highly perishable, necessitating post-harvest processing to extend shelf life and improve palatability.

In many regions of China, mature *T. sinensis* leaves are dried and brewed into a functional tea intended to alleviate intestinal inflammation. Conventional hot air-dried products often have a strong bitter taste and weak aroma, limiting consumer acceptance. Importantly, processing technologies can profoundly reshape the phytochemical profile and biological activities of plant materials (11). In black tea production, enzymatic oxidation (“fermentation”) converts simple catechins into theaflavins and thearubigins. Compared with their precursors, these compounds exhibit enhanced anti-inflammatory and gut microbiota-modulating properties (12, 13). Our group previously applied black tea processing (withering, rolling, enzymatic oxidation, drying) to *T. sinensis* leaves and observed increased flavonoids, limonoids, and amino acids, along with enhanced hypoglycemic activity (14).

However, it remains unclear whether conventional hot-air drying and black tea processing differentially influence the UC-protective effects of T. sinensis leaves. Furthermore, the underlying mechanisms related to intestinal barrier function, gut microbiota, and metabolic reprogramming have not been systematically investigated. Gut microbiota dysbiosis—a core feature of UC—manifests as reduced microbial diversity, loss of beneficial bacteria, and overgrowth of pro-inflammatory pathobionts (15). Restoring microbial homeostasis and enriching short-chain fatty acid (SCFA)-producing bacteria are critical therapeutic targets. Concurrently, downregulation of tight junction proteins (e.g., ZO-1, Occludin) disrupts intestinal barrier function, promoting luminal antigen translocation and exacerbating mucosal inflammation (16). Multi-omics approaches, including 16S rRNA sequencing and untargeted metabolomics, are powerful tools to elucidate host-microbe interactions and the molecular mechanisms of dietary interventions against colitis. This study aimed to determine whether black tea-processed T. sinensis leaves (TLT) alleviate DSS-induced ulcerative colitis in mice by enriching flavonoids and modulating gut microbiota. To this end, we compared TLT with conventional hot air-dried leaves (TL) and Keemun black tea (BT) as dietary controls, and evaluated colonic histopathology, intestinal barrier integrity, pro-inflammatory cytokines, gut microbiota composition, and leaf metabolomic profiles, followed by multi-omics integration analysis.

## 2 Materials and methods

### 2.1 Preparation of *T. sinensis* samples

Fresh *T. sinensis* leaves (var. ‘Heiyouchun') were harvested in May 2024 from a nursery (33.13°N, 115.66°E) in Taihe County, Anhui Province, China. The variety is officially certified as a National Agricultural Geographical Indication (AGI02871) and an improved forest tree variety of Anhui Province (Wan S-SV-TS-007-2019). Its identity as *Toona sinensis* was further confirmed by Prof. Sui Juanjuan (Fuyang Normal University) based on morphological characteristics. A voucher specimen has been deposited at the Herbarium of Fuyang Normal University (voucher No. FYNU-2019-042); this specimen was originally collected in 2019 from the same certified breeding base and represents the identical variety (‘Heiyouchun') used in the present study. For each biological replicate, mature leaves were collected from the seventh node of branches from three independent trees and pooled to minimize individual variation. Five biological replicates were prepared for each treatment group. Two processed samples were prepared as previously described (14) and are illustrated in Supplementary Figure S1: traditionally dried leaves (TL): fresh leaves were washed, drained, dried at 110 °C for 2 h, and cooled for 30 min. Processed *T. sinensis* tea (TLT): following black tea manufacturing procedures, fresh leaves were withered (25 °C, 12 h), rolled (60 min), fermented (28 °C, 85% RH, 6 h), and dried at 110 °C for 2 h. Keemun black tea (BT) was purchased from Anhui Guorun Tea Industrial Co., Ltd (Qimen, Anhui, China). All samples were ground into a fine powder (60-mesh sieve) and stored at −20 °C until further use.

### 2.2 Analysis of biochemical components

The contents of total flavonoids, total polyphenols, soluble sugars, free amino acids, and soluble protein in all samples (TL, TLT, and BT) were determined using standard colorimetric methods. All results were expressed on a dry weight basis (mg/g DW) to ensure consistency. For each analysis, powdered samples (0.5 g) were extracted with 10 ml of 70% aqueous methanol by ultrasonication for 30 min at room temperature, followed by centrifugation at 3,500 rpm for 10 min. The supernatant was used for the following determinations. Total flavonoid content was measured by the aluminum nitrate colorimetric assay at 510 nm, with rutin as the reference standard (17). The reaction mixture contained 1 ml of extract, 0.3 ml of 5% NaNO_2_, 0.3 ml of 10% Al(NO_3_)_3_, and 2 ml of 1 M NaOH, which was then made up to 10 ml with deionized water and incubated at room temperature for 15 min. Total polyphenols were determined using the Folin–Ciocalteu method at 765 nm, with gallic acid as the standard (18). Briefly, 0.5 ml of extract was mixed with 1 ml of Folin–Ciocalteu reagent and 3 ml of 10% Na_2_CO_3_, adjusted to a final volume of 10 ml with deionized water, then it was kept at room temperature in the dark for 2 h. Soluble sugars were quantified by the anthrone method at 620 nm, using glucose as the standard (19). An aliquot of 0.2 ml extract was reacted with 5 ml of anthrone reagent (0.2% in concentrated H_2_SO_4_) at 95 °C for 10 min. Free amino acids were assayed by the ninhydrin colorimetric method at 570 nm, with glycine as the standard. The extract (1 ml) was mixed with 1 ml of ninhydrin reagent and heated in a boiling water bath for 15 min. Soluble proteins were determined by the Bradford method at 595 nm, using bovine serum albumin (BSA) as the standard. A 0.1 ml aliquot of extract was added to 3 ml of Coomassie Brilliant Blue G-250 solution, and the mixture was made up to 10 ml with deionized water and incubated for 5 min. All spectrophotometric readings were conducted in triplicate on a Shimadzu UV-2,600 spectrophotometer with appropriate blank subtractions. Calibration curves were constructed using serial dilutions of the respective standards (concentration ranges and regression equations provided in Supplementary Table S1)

### 2.3 Animal experiment design

Forty male ICR mice (6 weeks old, 28–30 g) were purchased from Ziyuan Co., Ltd. (Hangzhou, China, SCXK2024-0004) and housed under specific pathogen-free conditions with *ad libitum* access to standard chow (12% energy from fat, 3.6 kcal/g, Supplementary Table S2) and water. Following 7 days of acclimatization, mice were divided into five groups (*n* = 8 per group): ([1) CK (blank control): normal drinking water; ([2) MD (DSS model): drinking water containing 3% (w/v) dextran sulfate sodium (molecular weight 36–50 kDa, MP Biomedicals); ([3) TL (traditionally dried leaves): DSS + TL infusion; ([4) TLT (processed tea): DSS + TLT infusion; ([5) BT (Keemun black tea control): DSS + BT infusion.

**Figure 1:**
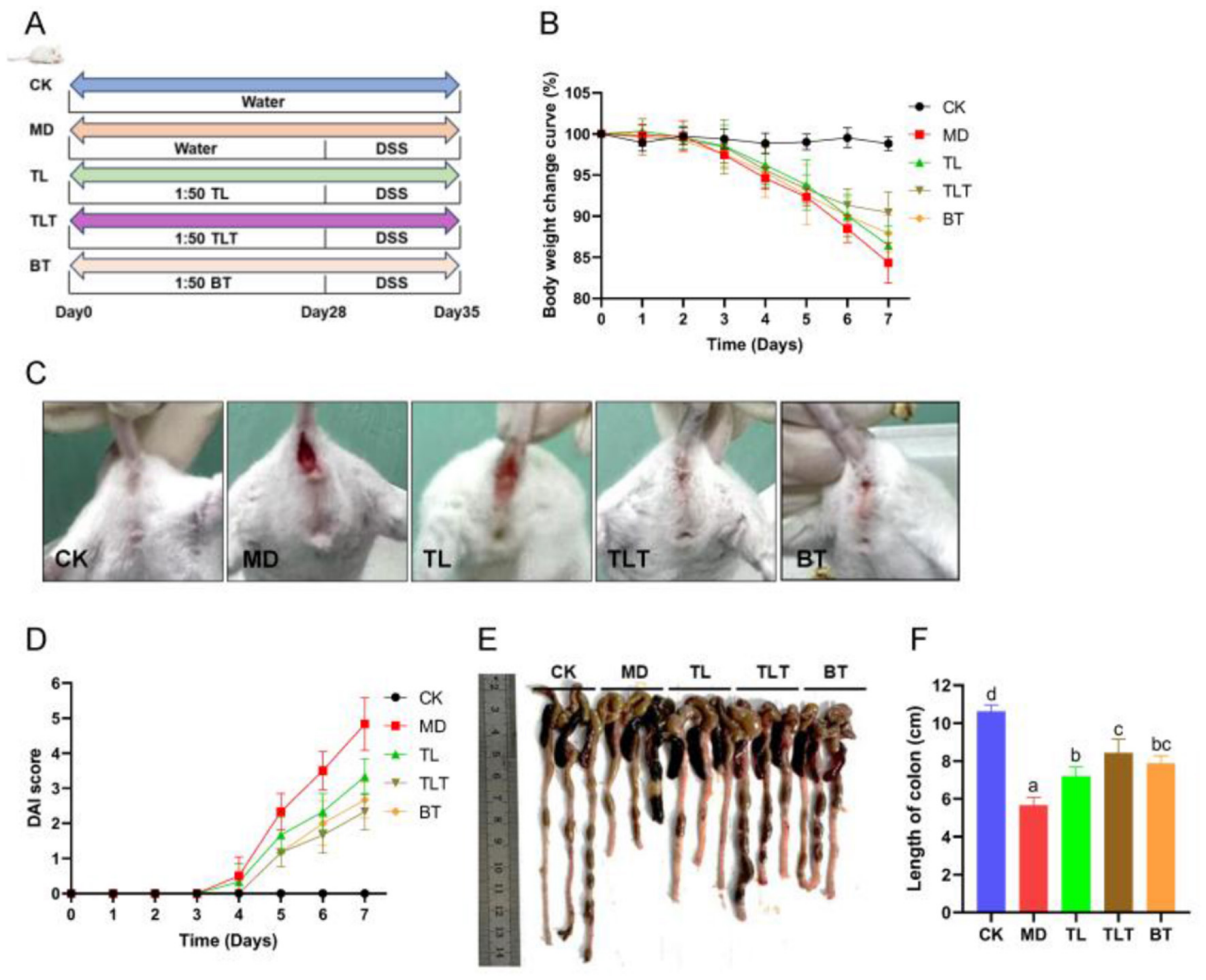
Different processing methods improve the anti-colitis effects of *T. sinensis* leaves. **(A)** Schematic of the experimental design. **(B)** Changes in body weight within 7 days. **(C)** Representative images of bloody stools. **(D)** Daily DAI scores. **(E)** Gross colon morphology of each group. **(F)** Statistical analysis of colon length. Groups: CK, blank control; MD, DSS-induced colitis model; TL, traditionally dried *T. sinensis* leaves; TLT, processed *T. sinensis* tea; BT, Keemun black tea control. Data are presented as mean ± SEM (*n* = 8). Different lowercase letters indicate *p* < 0.05.

Tea infusions were freshly prepared by steeping 1 g of leaves in 50 ml of boiling water for 10 min, followed by filtering. The resulting solution (20 mg/ml) was administered *ad libitum* as drinking water for 5 weeks. During the first 4 weeks, mice received tea infusions without DSS challenge. Subsequently, acute colitis was induced during the 5th week by adding 3% DSS (w/v) to the drinking water for 7 consecutive days, while tea infusion administration continued throughout this period (Figure 1A). Based on an average mouse body weight of ~30 g and daily water intake of ~5 ml (Supplementary Figure S2), the approximate daily dose was 3.33 g/kg body weight. Based on body surface area normalization, this corresponds to approximately 15 g/day (~5–6 cups) for a 60 kg human, representing the higher end of typical human consumption.

Body weight was recorded daily, and the disease activity index (DAI) was scored based on body weight loss, stool consistency, and rectal bleeding, as previously described (20). DAI scoring criteria are detailed in Supplementary Table S3. At the end of the experiment (day 7 of DSS treatment), mice were euthanized by cervical dislocation under anesthesia. Colon tissues were collected, measured for length, and either fixed in 4% paraformaldehyde for histopathological analysis or stored at −80 °C for subsequent assays (21). Colon contents were collected for gut microbiota analysis. All animal procedures were approved by the IACUC of Fuyang Normal University (protocol: FYNU2024AC011).

### 2.4 Histopathological analysis

Histological analysis followed a previously established procedure (22). Colon tissues were fixed in 4% paraformaldehyde, embedded in paraffin, sectioned (5 μm), and stained with hematoxylin and eosin (H&E). Histopathological changes (mucosal erosion, inflammatory cell infiltration, crypt damage) were observed under a light microscope (Olympus CX43, Tokyo, Japan) at 100 × magnification. The total histological injury score was evaluated based on the severity of inflammation, crypt damage, and mucosal ulceration (0–4 scale for each parameter; total score 0–12) as described (23). The scoring criteria are detailed in Supplementary Table S4. Crypt depth was measured using ImageJ (NIH, Bethesda, MD, USA).

### 2.5 Immunofluorescence staining

ZO-1 and Occludin expression on colon paraffin sections was detected by immunostaining as reported previously (21). Tissue sections were deparaffinized, rehydrated, antigen retrieved, and blocked with 5% BSA for 1 h at room temperature. Primary antibodies against ZO-1 (1:200, GB15195, Servicebio) and Occludin (1:500, GB111401, Servicebio) were applied overnight at 4 °C, followed by Cy3-conjugated goat anti-rabbit IgG (H+L) (1:300, Servicebio) for 1 h at room temperature. After DAPI counterstaining, fluorescence images were captured at 200 × magnification (scale bar = 50 μm) using a Leica SP8 confocal laser scanning microscope (Leica, Wetzlar, Germany), and intensity was quantified with ImageJ.

### 2.6 Measurement of pro-inflammatory cytokines

Blood samples were collected into heparinized tubes and centrifuged at 1,000 × g for 15 min at 4 °C to obtain plasma. The plasma supernatant was carefully transferred to fresh tubes and stored at −80 °C until analysis. Plasma levels of IL-1β and TNF-α were measured using commercial ELISA kits (Jianglai Biotech) per the manufacturer's instructions, and the absorbance was read using a Thermo Scientific Multiskan FC microplate reader.

### 2.7 Gut microbiota analysis

Total microbial DNA was extracted from cecal contents with the QIAamp DNA Stool Mini Kit (Qiagen, Hilden, Germany). The V3-V4 hypervariable region of the 16S rRNA gene was amplified with the primers 341F 5′-CCTAYGGGRBGCASCAG3′) and 806R 5′-GGATACNNGGGTATCTAAT3′). PCR products were purified and sequenced on the Illumina NovaSeq 6,000 platform (Illumina, San Diego, CA, USA). Raw reads were processed with QIIME2 (v2022.2). Alpha diversity indices (Chao1, Shannon, and observed features) were calculated for microbial richness and diversity, while beta diversity was assessed by principal coordinate analysis (PCoA) based on unweighted and weighted UniFrac distances. Relative abundance of bacterial taxa was quantified at phylum and genus levels. Functional potential was predicted using PICRUSt2 (v2.5.0) (24). MetaCyc pathway abundances were Z-scored and compared among groups.

### 2.8 Untargeted metabolomics analysis

Untargeted metabolomics data of TL and TLT samples were obtained from our previously published study (14). Chromatographic separation was performed on an Ultimate HPLC system (Thermo Fisher Scientific) equipped with an ACQUITY HSS T3 column (100 mm × 2.1 mm, 1.8 μm; Waters), and mass spectrometric detection was carried out on a Q Exactive Focus Orbitrap MS system (Thermo Fisher Scientific) with a heated electrospray ionization source. In the present study, these data were re-analyzed to identify differential metabolites associated with the anti-colitis effects of processed *T. sinensis* leaves. Raw data were processed using XCMS Online for peak deconvolution, alignment, and feature detection. Criteria for identifying differential metabolites between TL and TLT were as follows: relative standard deviation (RSD) < 30%, variable importance in projection (VIP) score > 1.0, |log_2_ (fold change)| > 1.0, and *p* < 0.05. A total of 23 key anti-inflammatory differential metabolites were selected. Detailed UHPLC-Orbitrap-MS/MS acquisition parameters were described in our previous report (14).

### 2.9 Statistical analysis

All data are presented as mean ± SEM (*n* = 8). One-way ANOVA followed by Tukey's *post hoc* test was used for multiple comparisons, with different lowercase letters indicating significant differences at *p* < 0.05.

## 3 Results

### 3.1 Different processing methods improve the anti-colitis effects of *T. sinensis* leaves

To evaluate the protective effects of differently processed *T. sinensis* leaves against DSS-induced colitis, mice received tea infusions *ad libitum* for 4 weeks followed by 7 days of 3% DSS challenge (Figure 1A). As shown in Figure 1B, DSS administration in the MD group induced steady body-weight loss starting on day 3, reaching approximately 15% by day 7. Both TL and TLT significantly reduced weight loss. Importantly, TLT led to significantly better recovery than TL (*p* < 0.05) and a slight, non-significant advantage over BT (*p* > 0.05). Representative images of bloody stools (Figure 1C) visually confirmed that TLT nearly eliminated rectal bleeding. The DAI score (Figure 1D), which integrates weight loss, stool consistency, and bleeding, was significantly elevated in MD mice (DAI ≈ 5) but markedly reduced in TL and TLT groups. Notably, TLT reduced DAI to levels comparable to the BT control group (*p* > 0.05). Colon shortening, a hallmark of UC severity, was pronounced in MD mice (approximately 5.67 cm), whereas TL (approximately 7.20 cm), TLT (approximately 8.43 cm), and BT (approximately 7.86 cm) significantly restored colon length (Figures 1E, F; all *p* < 0.05 vs. MD). Overall, TLT showed marginally greater anti-colitis efficacy than BT and significantly better efficacy than TL. These findings indicate that black tea processing significantly improves the anti-colitis activity of *T. sinensis* leaves.

### 3.2 Processing ameliorates colonic histopathological damage in mice supplemented with *T. sinensis* leaves

**Figure 2:**
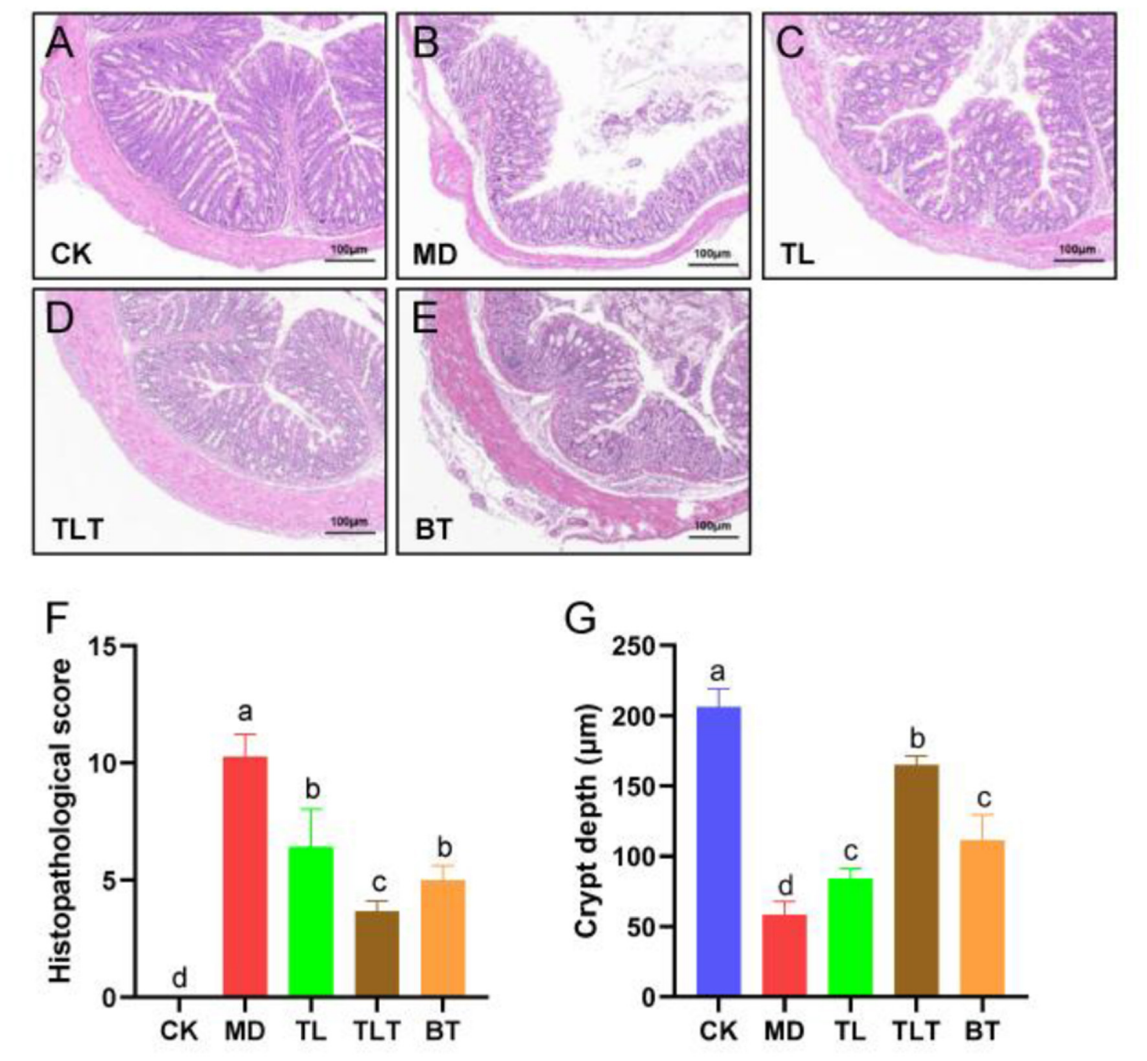
Processing ameliorates colonic histopathological damage in mice supplemented with *T. sinensis* leaves. **(A–E)** Representative H&E-stained colon sections (100 × magnification). **(F)** Total histological injury score. **(G)** Crypt depth quantification. Different letters denote significant differences (*p* < 0.05).

H&E-stained colon sections (Figures 2A–E) revealed severe mucosal erosion, crypt loss, inflammatory cell infiltration, and edema in MD mice. TL and BT treatment partially restored epithelial architecture, but residual crypt distortion remained. Remarkably, TLT almost completely preserved crypt integrity and goblet cell populations. The total histological injury score (Figure 2F) was highest in MD (10.26 ± 0.42), significantly reduced by TL (6.42 ± 0.71), and further reduced by TLT (3.66 ± 0.20, *p* < 0.05 vs. TL), with TLT also showing a slight improvement over BT (4.98 ± 0.28). Regarding crypt depth (Figure 2G), a similar trend was observed, with the TLT group exhibiting significantly greater colon crypt depth than both the TL and BT groups (*p* < 0.05). These data indicate that processed *T. sinensis* tea confers superior histoprotection.

### 3.3 Processed *T. sinensis* leaves regulate intestinal barrier integrity and inflammatory response

**Figure 3:**
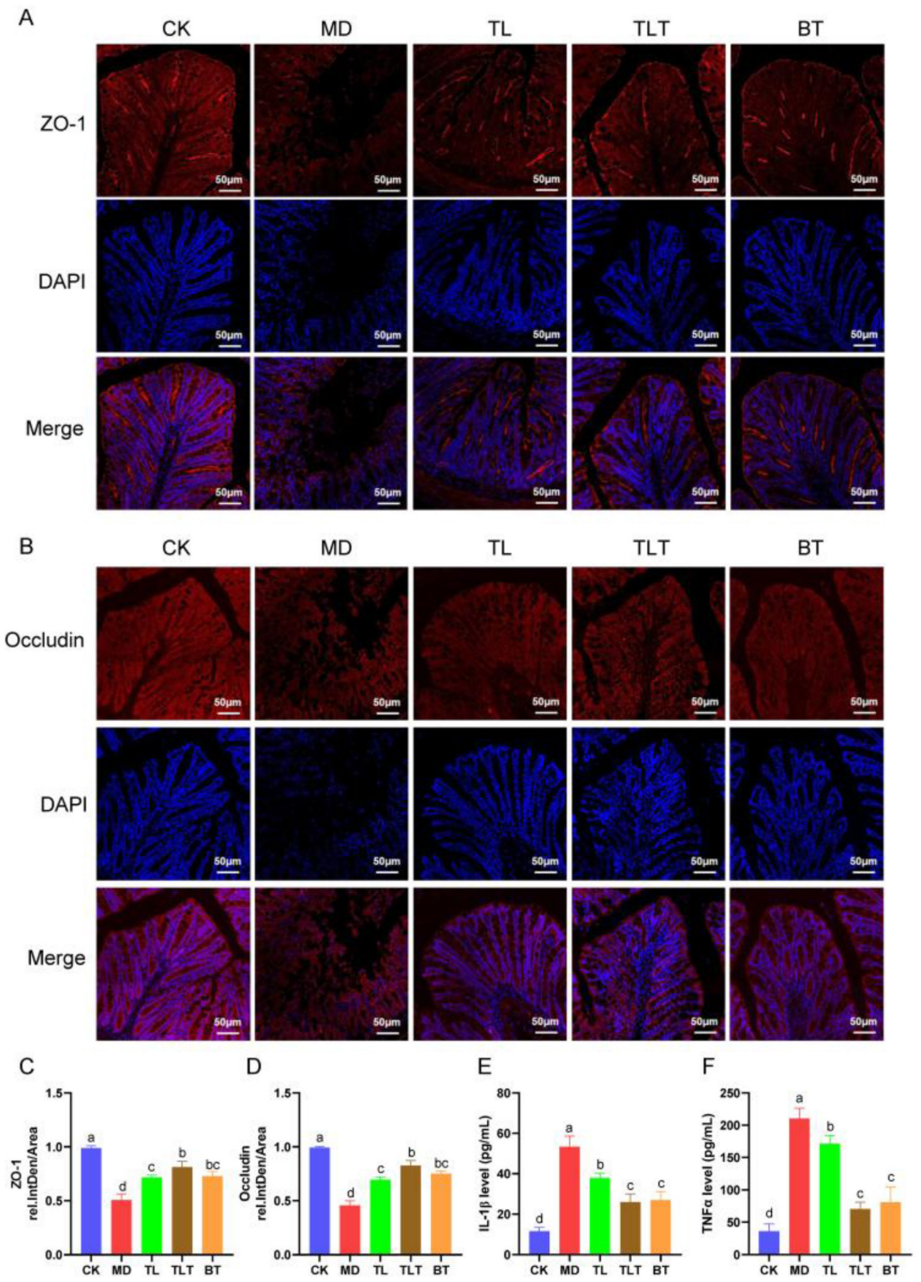
Processed *T. sinensis* leaves regulate intestinal barrier integrity and inflammatory response. **(A, B)** Immunofluorescence staining of ZO-1 and Occludin in colon tissues; scale bar = 50 μm, 200 × magnification. **(C, D)** Fluorescence intensity of ZO-1 and Occludin. **(E, F)** Colonic levels of pro-inflammatory IL-1β and TNF-α. Significant differences were marked by different letters (*p* < 0.05).

Immunofluorescence staining (Figures 3A, B) showed that DSS disrupted the continuous localization of tight junction proteins ZO-1 and Occludin at intercellular borders, with markedly reduced fluorescence intensity (Figures 3C, D). Both TL and TLT restored ZO-1 and Occludin expression, with TLT achieving a significantly greater increase than TL (*p* < 0.05) and a slightly better effect than BT. In parallel, colonic pro-inflammatory cytokines IL-1β and TNF-α (Figures 3E, F) were dramatically elevated in MD mice (approximately 53.32 pg/mg and 210.49 pg/mg, respectively). TL reduced these cytokines by approximately 29.03% and 18.56%, whereas TLT suppressed IL-1β and TNF-α by approximately 51.37% and 66.58% (*p* < 0.01 vs. MD), showing no significant difference from BT but with a modest numerical advantage. Overall, TLT demonstrated slightly superior efficacy in restoring gut barrier function and suppressing local inflammation compared to BT, and significantly better efficacy than TL.

### 3.4 Biochemical components of processed *T. sinensis* leaves correlate with protective effects

**Figure 4:**
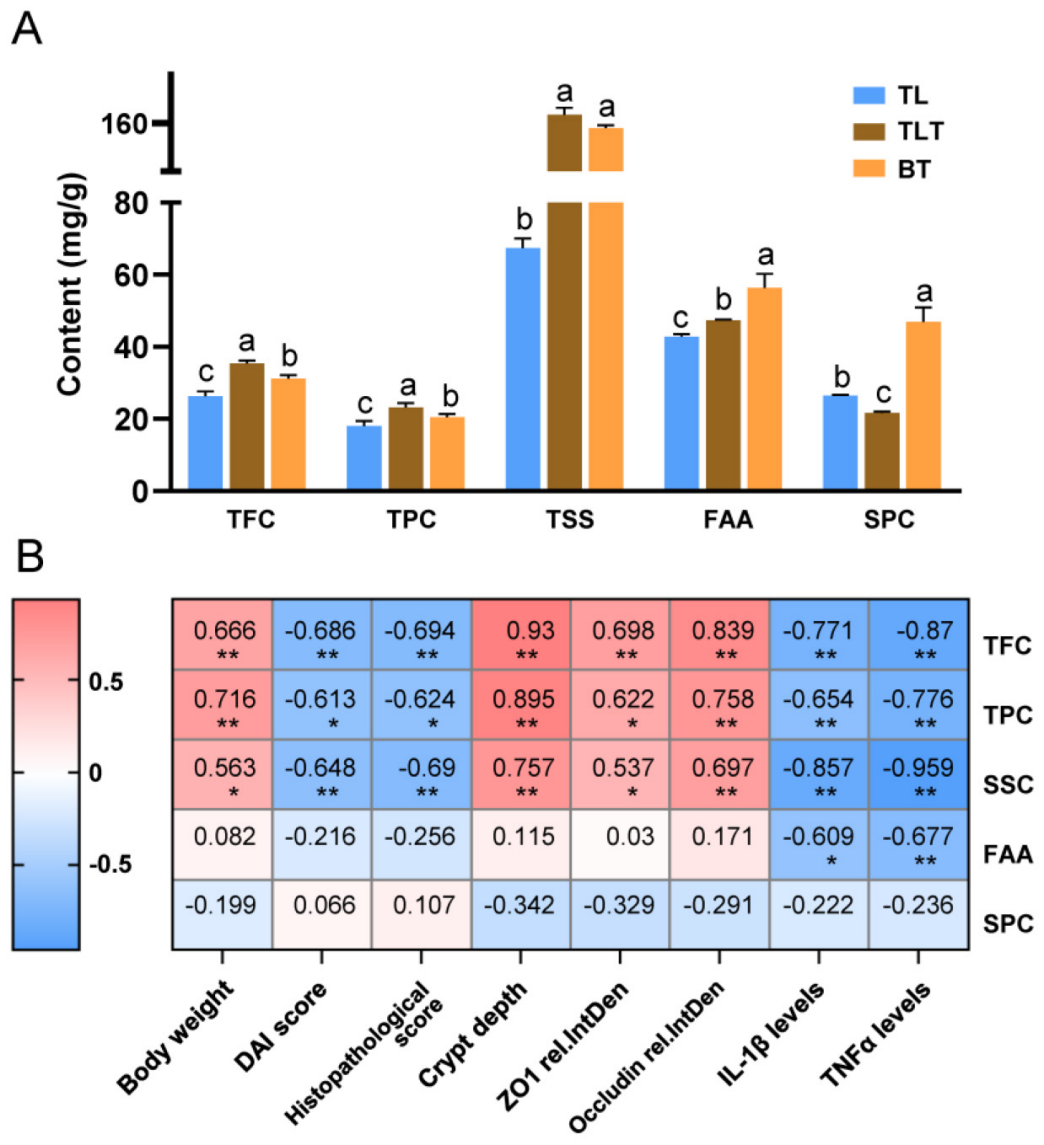
Biochemical components of processed *T. sinensis* leaves and their correlation with colitis protective effects. **(A)** Contents of total flavonoids (TFC), total polyphenols (TPC), soluble sugars (SSC), free amino acids (FAA) and soluble protein (SPC) in different processed leaves (all values expressed as mg/g dry weight, DW). **(B)** Spearman correlation heatmap between biochemical components and protective indicators. Red represents positive correlation, and blue represents negative correlation.

Quantification of bioactive components (Figure 4A) showed that TLT possessed significantly higher total flavonoids (35.48 ± 0.41 mg/g DW) than both TL (26.30 ± 0.78 mg/g DW) and BT (31.2 ± 0.55 mg/g DW) (*p* < 0.05). Total polyphenol levels were also markedly higher in TLT (23.26 ± 0.62 mg/g DW) compared with TL (18.03 ± 0.80 mg/g DW) and BT (20.5 ± 0.51 mg/g DW) (*p* < 0.05). Soluble sugars were remarkably elevated in TLT (163.5 ± 1.69 mg/g DW), showing no significant difference from BT (158.0 ± 1.21 mg/g DW, *p* > 0.05) and being significantly higher than TL (67.33 ± 1.56 mg/g DW, *p* < 0.05). Free amino acids were higher in BT (56.43 ± 2.19 mg/g DW) than in TLT (47.36 ± 0.12 mg/g DW) and TL (42.87 ± 0.36 mg/g DW) (*p* < 0.05). Soluble protein content was the lowest in TLT (21.71 ± 0.22 mg/g DW), significantly lower than that in both TL (26.50 ± 0.08 mg/g DW) and BT (46.93 ± 2.28 mg/g DW) (*p* < 0.05).

Spearman correlation analysis (Figure 4B) revealed that total flavonoids and total polyphenols were strongly positively correlated with protective endpoints such as body weight, crypt depth, and tight junction protein expression (|r| > 0.6, *p* < 0.05). Conversely, they showed strong negative correlations with DAI score, histopathological score, and pro-inflammatory cytokines (IL-1β and TNF-α) (|r| > 0.6, *p* < 0.05). Soluble sugars also exhibited strong to moderate correlations with these protective indices, particularly negative correlations with IL-1β (r = −0.857) and TNF-α (r = −0.959). In contrast, free amino acids and soluble protein showed relatively weak or inconsistent correlations with the assessed endpoints. These results indicate that black tea processing enhances key bioactive components, particularly total flavonoids and total polyphenols, which directly underpin the improved anti-colitis efficacy.

### 3.5 Different processing reshapes gut microbiota composition in colitis mice

**Figure 5:**
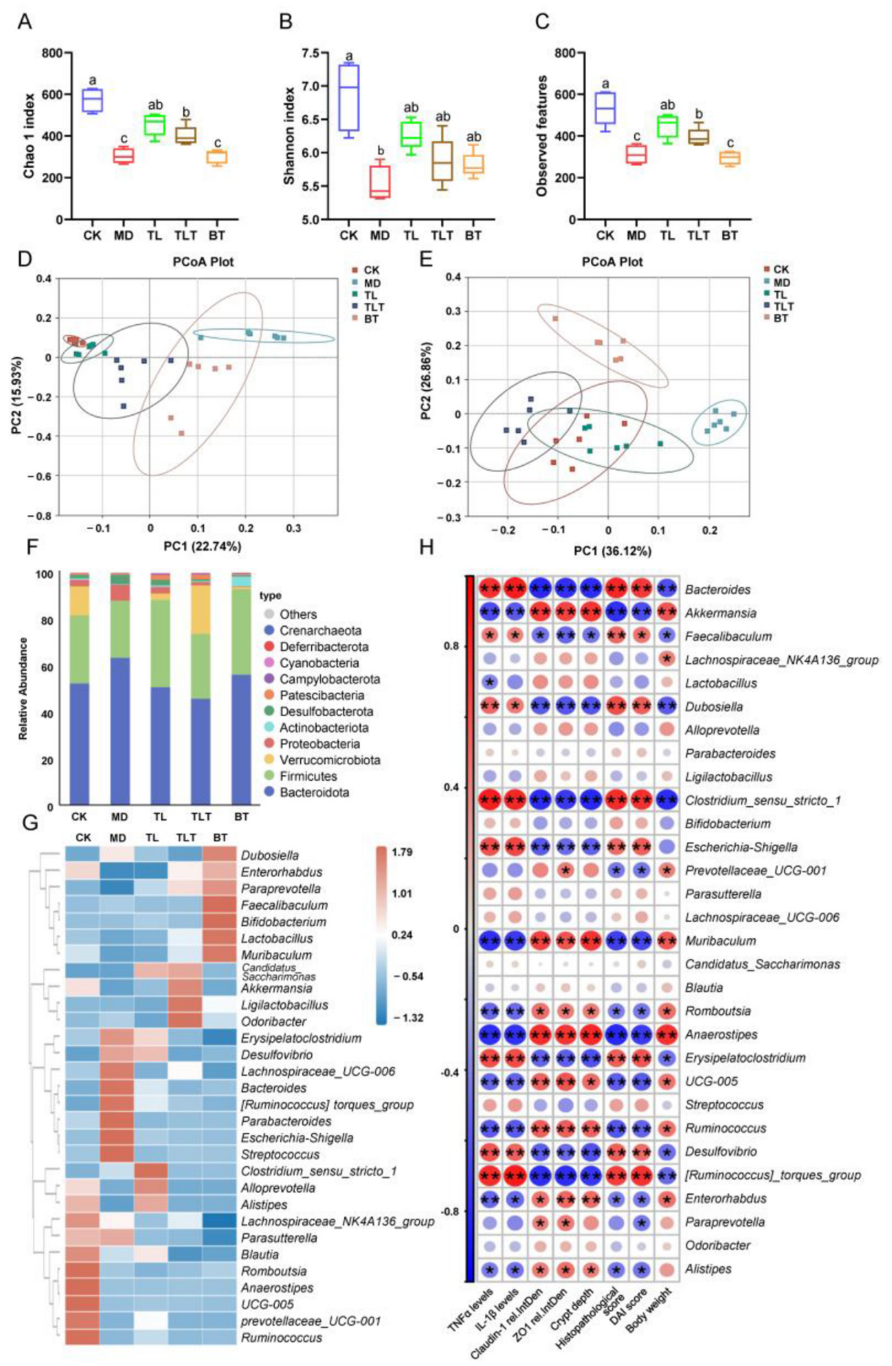
Different processing reshapes gut microbiota composition in colitis mice. **(A)** Chao 1 index. **(B)** Shannon index. **(C)** Number of observed bacterial features. **(D, E)** β-diversity via PCoA based on unweighted and weighted UniFrac distances. **(F)** Top 10 phylum-level bacterial abundance. **(G)** Heatmap of top 30 differential genera. **(H)** Spearman correlation between key genera and phenotypic indexes. Red: positive correlation; blue: negative correlation.

DSS-induced gut dysbiosis was characterized by markedly reduced alpha-diversity (Chao1, Shannon, observed features) in the MD group (Figures 5A–C). Both TL and TLT significantly increased the Chao1 and Shannon indices and restored microbial diversity relative to the MD group (*p* < 0.05), while BT showed a relatively weaker capacity to improve alpha-diversity. Beta-diversity analysis via PCoA (Figures 5D, E) showed clear separation between the CK and MD groups. By comparison, the TL and TLT clusters were closer to the CK group, indicating that processed *T. sinensis* leaves effectively facilitate the restoration of overall gut microbiota structure. At the phylum level (Figure 5F), DSS intervention elevated the relative abundance of Bacteroidota and reduced that of Firmicutes, leading to a decreased Firmicutes/Bacteroidota (F/B) ratio. Both TL and TLT reversed this microbial shift, with TLT exerting a more pronounced regulatory effect.

The heatmap of the top 30 differential genera (Figure 5G) further revealed distinct modulatory patterns across treatments. The TL group selectively enriched *Clostridium_sensu_stricto_1, Alloprevotella*, and *Alistipes*; the TLT group prominently enriched *Candidatus_Saccharimonas, Akkermansia, Ligilactobacillus*, and *Odoribacter*; while the BT group enriched *Dubosiella, Enterorhabdus, Paraprevotella, Faecalibaculum, Bifidobacterium, Lactobacillus*, and *Muribaculum*. In contrast, the MD group was characterized by the enrichment of inflammation-related genera, including *Erysipelatoclostridium, Desulfovibrio, Lachnospiraceae_UCG-006, Bacteroides*, [*Ruminococcus*]*_torques_group, Parabacteroides, Escherichia–Shigella*, and *Streptococcus*.

Spearman correlation analysis (Figure 5H) confirmed that beneficial genera including *Akkermansia, Muribaculum, Romboutsia, Anaerostipes, UCG-005, Ruminococcus, Enterorhabdus*, and *Alistipes* were significantly positively correlated with protective phenotypic indicators such as colon length and ZO-1 expression. Conversely, *Bacteroides, Faecalibaculum, Dubosiella, Clostridium_sensu_stricto_1, Escherichia–Shigella, Desulfovibrio*, and [*Ruminococcus*]*_torques_group* showed significant negative correlations with these protective endpoints. Collectively, both TL and TLT effectively restored microbial alpha-diversity, remodeled overall microbiota structure, and enriched beneficial bacterial taxa, thereby reshaping a balanced gut microbial ecosystem conducive to colitis alleviation. PICRUSt2 functional prediction further revealed that DSS-induced colitis (MD) suppressed pathways involved in nucleotide synthesis (e.g., PWY-7229, PWY-6126, PWY-5686) and SCFA precursor production (PWY-5100), while enhancing stress-associated pathways (P42-PWY, DTDPRHAMSYN-PWY) (Supplementary Figure S3). TLT treatment reversed these changes, restoring beneficial pathways to levels close to the CK group and reducing stress pathways.

### 3.6 Differential anti-inflammatory metabolites of *T. sinensis* leaves induced by processing

**Figure 6:**
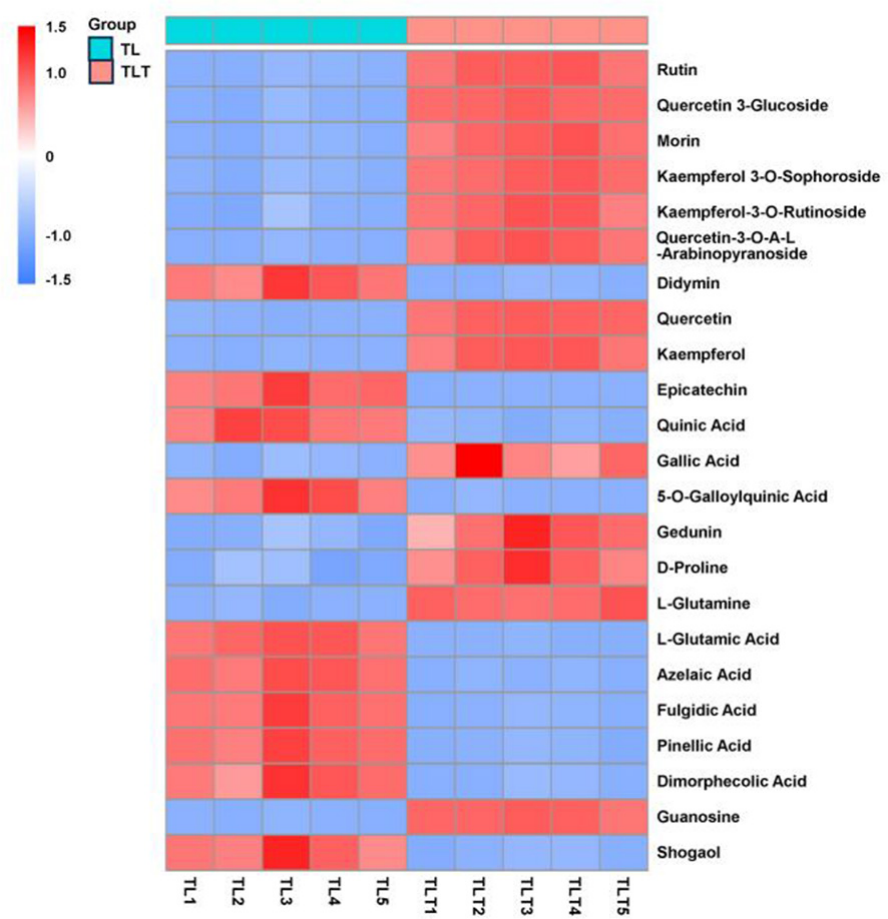
Heatmap of 23 key anti-inflammatory differential metabolites between TL and TLT. Data were re-analyzed from our previously published untargeted metabolomics data (14). Red and blue indicate higher and lower metabolite abundance, respectively.

Untargeted metabolomics was applied to screen differential metabolites between TL and TLT, with screening thresholds set as VIP > 1.0, *p* < 0.05 (nonparametric test), and fold change > 2.0 or < 0.5. In total, 23 key anti-inflammatory differential metabolites were identified, including 9 flavonoids and flavonoid glycosides, 1 catechin, 3 phenolic acids, 1 limonoid, 3 amino acids, 4 organic acids, and 2 other compounds (Figure 6, Supplementary Table S5).

Compared with TL, most flavonoids and flavonoid glycosides were markedly upregulated in TLT. The most prominent increases were observed for quercetin (11.73-fold), kaempferol (4.67-fold), kaempferol 3-O-sophoroside (4.41-fold), and rutin (3.92-fold). Gallic acid (2.12-fold) and the limonoid gedunin (3.36-fold) were also significantly enriched in TLT. By contrast, several compounds were downregulated in TLT, such as didymin (0.34-fold), epicatechin (0.16-fold), azelaic acid (0.37-fold), and fulgidic acid (0.34-fold). Additionally, guanosine (5.60-fold), D-proline (2.28-fold), and L-glutamine (2.76-fold) were significantly upregulated in TLT.

These differential metabolites, especially the enriched flavonoids and phenolic acids, have been well documented to suppress NF-κB signaling and strengthen intestinal barrier function (25, 26), providing a critical molecular explanation for the superior anti-colitis capacity of TLT.

### 3.7 Integrated correlation analysis of metabolites, gut microbiota, and protective endpoints

**Figure 7:**
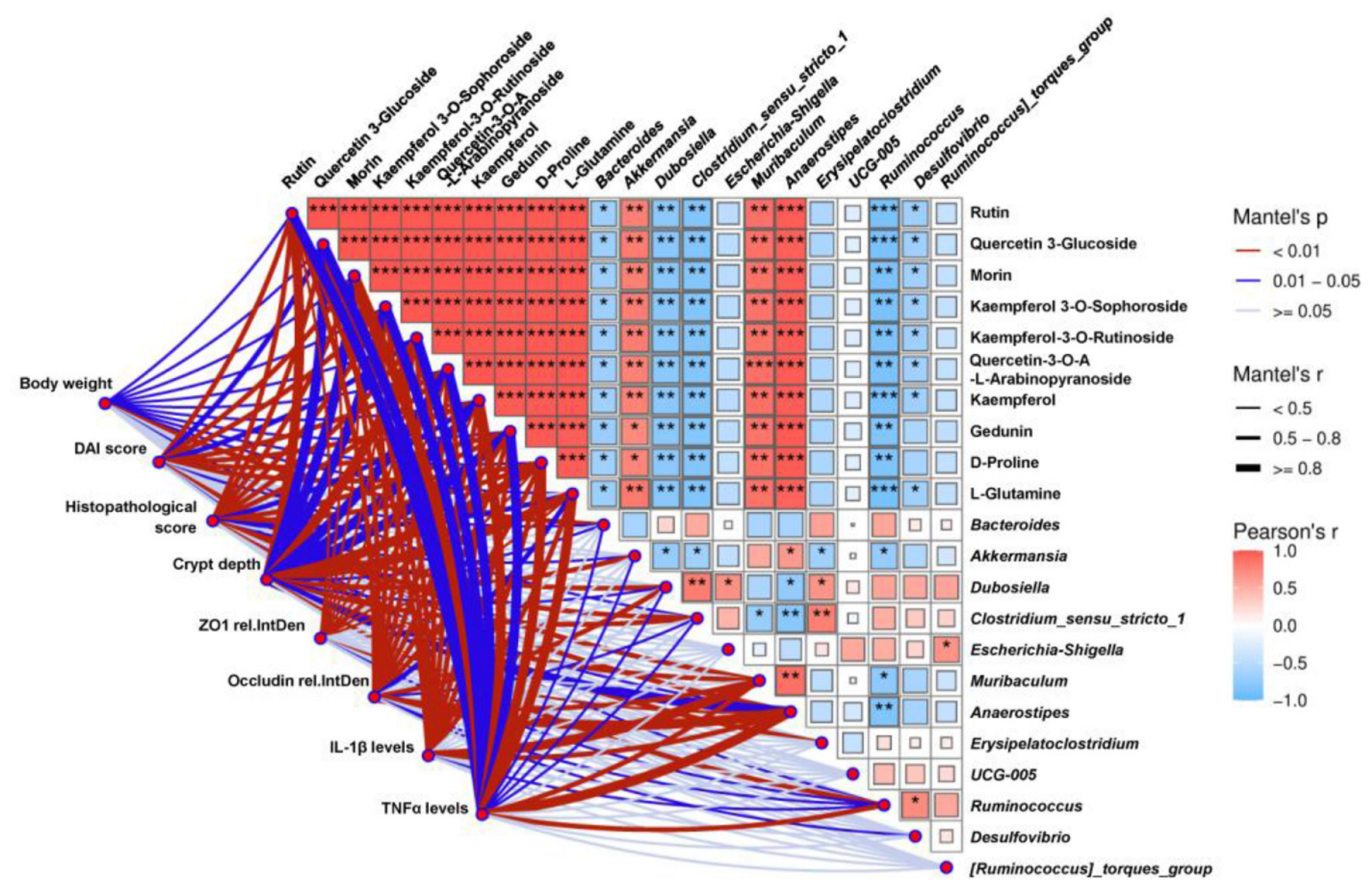
Integrated correlation analysis of metabolites, gut microbiota and protective endpoints. Line color indicates correlation *p*-value, and line thickness represents correlation strength. Square color reflects correlation direction: red for positive and blue for negative correlations, while square size indicates correlation magnitude.

Multi-omics integrated correlation analysis (Figure 7) constructed a regulatory network among differential metabolites, pivotal bacterial genera, and phenotypic protective endpoints. To clarify the underlying associations, we selected the top 10 differential metabolites and 12 key gut microbial genera that showed significant correlations with colitis-related protective indices. Further correlation analysis revealed that these 10 core metabolites were significantly positively correlated with *Akkermansia, Muribaculum*, and *Anaerostipes*, whereas they exhibited marked negative correlations with *Bacteroides, Dubosiella, Clostridium_sensu_stricto_1, Ruminococcus*, and *Desulfovibrio*. These results imply a synergistic interaction between characteristic metabolites and beneficial gut microbes in regulating host physiological homeostasis. Collectively, processing-induced metabolic reprogramming coordinately modulates gut microbiota composition and host inflammatory responses, thereby alleviating DSS-induced colitis. This metabolite–microbiota crosstalk also provides a promising targeted strategy for the intervention of inflammatory bowel diseases.

## 4 Discussion

Based on current evidence, this study provides the first comparative evidence that black tea processing enhances the anti-UC efficacy of *T. sinensis* leaves relative to conventional hot air drying. This improved efficacy is primarily attributed to processing-driven biochemical transformations. TLT exhibited 29–35% higher total polyphenol and flavonoid contents than TL from identical raw materials, likely due to processing-induced cell-wall disruption, which releases bound phenolics (27). Strong correlations between bioactive components and phenotypic markers (e.g., |r| > 0.8 for flavonoids vs. TNF-α) further confirm their protective functions. Notably, kaempferol and quercetin glycosides—two compounds markedly enriched in TLT—are known to block NLRP3 inflammasome activation and enhance Occludin expression (28–31). Similar protective effects have been reported for other flavonoid-rich plants, such as mulberry anthocyanins (32) and green tea polyphenols (33). Notably, TLT restored colon length from 5.67 cm to 8.43 cm, an effect that may involve the synergistic action of multiple *Toona*-specific flavonoids. In contrast, conventional drying lacks rolling, enzymatic oxidation, and cell wall degradation, limiting the release of bound phenolics and likely explaining the weaker efficacy of TL.

Gut microbiota remodeling also contributes to TLT-mediated colitis mitigation. DSS-induced dysbiosis typically depletes short-chain fatty acid (SCFA)-producing bacteria and promotes pro-inflammatory pathogens (34). TLT treatment selectively enriched beneficial commensals, including *Akkermansia, Odoribacter, Ligilactobacillus, and Candidatus_Saccharimonas*. These bacteria are key SCFA producers; SCFAs serve as the preferred energy source for colonocytes, enhance intestinal barrier function, and mediate anti-inflammatory effects via the GPR43 signaling pathway (35, 36). The positive correlation between flavonoid glycosides and *Akkermansia* suggests a prebiotic role for TLT, consistent with reports that dietary polyphenols selectively promote beneficial gut microbes (25, 37). Additionally, TLT suppressed the abundance of *Escherichia–Shigella* and *Streptococcus*—pathobionts closely linked to UC exacerbation—thereby reducing endotoxin-induced TNF-α overproduction (32, 38). Unlike BT, which strongly enriched classical *Lactobacillus*, TLT moderately increased *Lactobacillus* abundance while enriching *Ligilactobacillus*, a related genus known to produce SCFAs and antimicrobial peptides that strengthen the intestinal barrier and inhibit pathogens (39). This differential modulation likely reflects distinct phytochemical profiles: BT provides theaflavins, whereas TLT contains *Toona*-specific flavonoids (e.g., kaempferol, quercetin glycosides) that may preferentially support *Ligilactobacillus*. Thus, TLT exerts a microbiota modulation pattern distinct from classic black tea, contributing to its unique anti-colitis efficacy.

PICRUSt2 functional prediction further supported these compositional changes. TLT treatment enriched pathways involved in SCFA precursor production (PWY-5100) and nucleotide biosynthesis, indicating enhanced microbial growth and beneficial metabolism. Conversely, the MD group exhibited elevated stress-associated pathways (P42-PWY, DTDPRHAMSYN-PWY), which are often linked to bacterial envelope stress and pathogenicity. The reduction of these pathways by TLT suggests alleviation of microbial stress, consistent with improved gut barrier function and reduced inflammation. Collectively, these functional data provide a mechanistic link between TLT-modulated gut microbiota and colonic protection.

Integrated multi-omics correlation analysis revealed that processing-induced metabolic alterations do not act independently but synergistically with gut microbiota to maximize intestinal protection. A virtuous regulatory network emerged linking anti-inflammatory metabolites, SCFA-producing bacteria, tight junction proteins, and pro-inflammatory cytokines. Specifically, phenolic and flavonoid components enriched SCFA-synthesizing bacteria, upregulated tight junction expression, and relieved inflammation. In turn, the improved mucosal microenvironment further facilitated microbial homeostasis. This systematic crosstalk suggests a plausible mechanistic model in which black tea processing transforms raw plant materials into functional foods with enhanced bioactivity. However, direct causal relationships between specific metabolites, bacterial taxa, and protective endpoints remain to be validated through targeted interventions (e.g., metabolite gavage or fecal microbiota transplantation).

Interestingly, TLT showed slightly superior efficacy to BT, a classic fermented tea widely recognized for its anti-inflammatory potential (40, 41). This may be explained by the distinct metabolic signatures between TLT and BT. BT accumulates tea-derived theaflavins from *Camellia sinensis*, whereas TLT contains both processing-induced flavonoid derivatives (primarily quercetin glycosides). Interestingly, BT enriched *Bifidobacterium* and *Lactobacillus*, which are classic probiotic genera associated with black tea consumption (42), whereas TLT uniquely enriched *Akkermansia* and *Odoribacter*. This distinct microbiota profile may explain the slightly superior efficacy of TLT in restoring colon length and reducing histological injury. These *Toona*-specific flavonoids, identified as key metabolites in our multi-omics network, may contribute to the enhanced efficacy of TLT. Collectively, fermented *T. sinensis* leaf tea holds promise as a novel regional functional food for UC adjunctive therapy. Nevertheless, whether the differential phytochemical profiles translate into divergent physiological functions requires further comparative metabolomics studies.

The major novel contributions of this study are threefold. First, it provides the first comparative evidence that black tea processing enhances the anti-UC activity of *T. sinensis* leaves relative to conventional hot air drying. Second, it integrates multi-omics data to reveal a coordinated metabolite–microbiota network underlying this enhanced efficacy, specifically identifying kaempferol and quercetin glycosides as key bioactive metabolites. Third, it demonstrates that TLT, a non-*Camellia* fermented tea, exhibits efficacy comparable to classic Keemun black tea, supporting the value-added utilization of underutilized medicinal-edible plants through controlled processing.

Several limitations require acknowledgment. First, the 7-day DSS model only recapitulates acute colitis; chronic recurrent colitis models are needed to assess long-term efficacy and biosafety of TLT. Second, the administered dosage (20 mg/ml) was based on previous studies (14); dose–response experiments are necessary to establish causality. Third, gut microbiota was analyzed only at the endpoint without a pre-colitis time point (e.g., week 4); therefore, whether TLT directly enriches beneficial commensals or acts indirectly via colitis alleviation remains unclear. Longitudinal sampling is needed to establish causality. Fourth, untargeted metabolomics was performed on leaf extracts rather than intestinal contents or feces; future *in vivo* metabolic profiling should examine the bioavailability and metabolic fate of key active constituents. Finally, rigorous clinical trials are essential to translate these preclinical findings into dietary applications. From a food processing perspective, our findings underscore the high-value potential of *T. sinensis* leaves. Although traditional hot air drying is straightforward, controlled black tea processing not only improves sensory qualities (aroma and taste) but also significantly enhances physiological activity. Future studies should focus on optimizing processing parameters—such as duration, temperature, and humidity—to specifically promote the accumulation of bioactive compounds. In the context of food security and human health, underutilized traditional medicinal-edible plants such as *T. sinensis* represent valuable plant-based food resources. Black tea processing not only improves sensory quality but also upgrades phytochemical profiles and nutritional functions, offering a feasible strategy for high-value conversion of agricultural by-products and development of natural functional foods for intestinal health management.

## 5 Conclusions

In summary, compared with conventional hot air drying, black tea processing significantly enhances the anti-UC activity of *T. sinensis* leaves. TLT alleviates DSS-induced colitis through multiple mechanisms: it elevates flavonoids and polyphenols—including the anti-inflammatory metabolites kaempferol and quercetin glycosides—restores intestinal barrier integrity (ZO-1, Occludin), suppresses pro-inflammatory cytokines (IL-1β, TNF-α), and enriches SCFA-producing gut microbiota such as *Akkermansia, Odoribacter, and Ligilactobacillus*. Multi-omics integration reveals coordinated metabolite–microbiota crosstalk supporting these protective effects. These findings provide a theoretical basis for value-added processing of *T. sinensis* leaves as a functional food for UC dietary intervention. Future studies should validate these effects in chronic colitis models and clinical settings, and optimize processing conditions to maximize bioactive compound accumulation.

## Data Availability

The original contributions presented in the study are publicly available. The raw data generated in this study can be found in the NCBI Sequence Read Archive (SRA) under BioProject accession number: PRJNA1481352.
