# Supplementary material for: Black tea-processed Toona sinensis leaves alleviate DSS-induced ulcerative colitis in mice by enriching flavonoids and modulating gut microbiota
Source: Front Nutr. 2026 Jul 14;13:1888003. doi: 10.3389/fnut.2026.1888003 (PMC13407273; doi:10.3389/fnut.2026.1888003)
Supplement: Supplementary file 1 [file Table_1.docx]

**Supplementary Material**

**Table S1** Linear equations and correlation coefficients (R^2^) of main active components

| Compound | Reference standard | Linear equation | R^2^ |
| --- | --- | --- | --- |
| Total flavonoids | Rutin | y = 0.0016x + 0.0515 | 0.998 |
| Total polyphenols | Gallic acid | y = 0.0113x + 0.0783 | 0.999 |
| Soluble sugar | Glucose | y = 3.7001x + 0.284 | 0.997 |
| Free amino acids | Glycine | y = 2.732x + 0.115 | 0.999 |
| Soluble protein | Bovine Serum Albumin | y = 0.204x + 0.284 | 0.998 |

Note: x, amount of each analyzed tea infusions (mg/mL); y, Abs.

**Table S2** The composition of the experimental diets

| **Ingredient (g)** | **Standard chow** |
| --- | --- |
| Casein | 191 |
| Cornstarch | 484 |
| Dextrin | 120 |
| Sucrose | 66 |
| Soybean oil | 24 |
| Lard | 19 |
| Cellulose | 48 |
| Mineral mix | 33 |
| Vitamin mix | 10 |
| L-Cystine | 3 |
| Line bitartrate | 2 |
| TBHQ | 0.01 |
| Total | 1000 |
| Total energy |  |
| Protein, % | 20.6 |
| Fat, % | 12.0 |
| Carbohydrate, % | 67.4 |
| Energy, Kcal/g | 3.616 |

**Table S3** Disease activity index (DAI) scoring standard

| Score | Weight loss (%) | Stool consistency | Gross bleeding |
| --- | --- | --- | --- |
| 0 | None | Normal | None |
| 1 | 1-5 | Loose stools | Hemoccult |
| 2 | 5-10 |  |  |
| 3 | 10-20 | Diarrhea | Gross blood |
| 4 | >20 |  |  |

**Table S4** Histological scoring criteria for colon sections

| Score | Inflammation severity | Inflammation extent | Crypt damage |
| --- | --- | --- | --- |
| 0 | None | None | None |
| 1 | Slight | Mucosal | Damage to the basal third of the crypt |
| 2 | Moderate | Submucosal | Damage to the basal two-thirds of the crypt |
| 3 | Severe | Transmural | Only surface epithelium intact |
| 4 |  |  | Loss of entire crypt and epithelium |

**Table S5** The 23 key anti-inflammatory differential metabolites of TL and TLT.

| **Compound Name** | **RT (min)** | **Adduct m/z** | **Formula** | **Ion mode** | **Fragments** | **VIP** | ***p*-Value** | **FC(TLT/TOL)** |
| --- | --- | --- | --- | --- | --- | --- | --- | --- |
| **Flavonoids and**  **flavonoid glycosides** |  |  |  |  |  |  |  |  |
| Rutin | 11.51 | 609.146 | C_27_H_30_O_16_ | [M-H]- | 609, 301, 300, 271, 151 | 8.53 | 1.00×10^−10^ | 3.92 |
| Quercetin 3-Glucoside | 11.85 | 463.0876 | C_21_H_20_O_12_ | [M-H]- | 463, 301, 300, 255, 151 | 10.85 | 5.00×10^−12^ | 2.97 |
| Morin | 11.86 | 303.0497 | C_15_H_10_O_7_ | [M+H]+ | 303, 257, 229, 153, 137 | 5.25 | 1.00×10^−9^ | 3.39 |
| Kaempferol 3-O-Sophoroside | 11.93 | 609.146 | C_27_H_30_O_16_ | [M-H]- | 609, 301, 300, 271, 151 | 1.84 | 5.00×10^−11^ | 4.41 |
| Kaempferol-3-O-Rutinoside | 12.13 | 593.1503 | C_27_H_30_O_15_ | [M-H]- | 593, 285, 255, 227 | 2.84 | 9.00×10^−9^ | 2.14 |
| Quercetin-3-O-Α-L-Arabinopyranoside | 12.30 | 433.0773 | C_20_H_18_O_11_ | [M-H]- | 433, 300, 271, 255 | 5.09 | 1.00×10^−9^ | 3.85 |
| Didymin | 14.32 | 593.1297 | C_28_H_34_O_14_ | [M-H]- | 447, 285 | 1.01 | 4.00×10^−8^ | 0.34 |
| Quercetin | 14.65 | 301.0352 | C_15_H_10_O_7_ | [M-H]- | 301, 178, 151, 107 | 4.85 | 5.00×10^−12^ | 11.73 |
| Kaempferol | 15.74 | 285.0404 | C_15_H_10_O_6_ | [M-H]- | 285, 151, 137, 93 | 2.30 | 4.00×10^−10^ | 4.67 |
| **Catechins** |  |  |  |  |  |  |  |  |
| Epicatechin | 9.52 | 289.0717 | C_15_H_14_O_6_ | [M-H]- | 245, 151, 137 | 1.32 | 8.00×10^−9^ | 0.16 |
| **Phenolic acids** |  |  |  |  |  |  |  |  |
| Quinic Acid | 1.38 | 193.0708 | C_7_H_12_O_6_ | [M+H]+ | 178, 133, 122 | 1.52 | 9.00×10^−6^ | 0.39 |
| Gallic Acid | 4.14 | 169.0144 | C_7_H_6_O_5_ | [M-H]- | 125, 127, 97 | 1.64 | 1.00×10^−7^ | 2.12 |
| 5-O-Galloylquinic Acid | 4.43 | 345.0819 | C_14_H_16_O_10_ | [M+H]+ | 153, 125 | 1.11 | 1.00×10^−7^ | 0.19 |
| **Limonoids** |  |  |  |  |  |  |  |  |
| Gedunin | 20.57 | 483.2376 | C_28_H_34_O_7_ | [M+H]+ | 423, 379, 161, 137 | 2.09 | 1.00×10^−5^ | 3.36 |
| **Amino acids** |  |  |  |  |  |  |  |  |
| D-Proline | 1.60 | 116.0706 | C_5_H_9_NO_2_ | [M+H]+ | 70 | 1.03 | 3.00×10^−6^ | 2.28 |
| L-Glutamine | 1.23 | 145.0619 | C_5_H_10_N_2_O_3_ | [M-H]- | 127, 109, 84, 41 | 1.77 | 3.00×10^−11^ | 2.76 |
| L-Glutamic Acid | 1.28 | 148.0604 | C_5_H_9_NO_4_ | [M+H]+ | 129, 84 | 3.64 | 1.00×10^−10^ | 0.32 |
| **Organic acids** |  |  |  |  |  |  |  |  |
| Azelaic Acid | 13.18 | 187.0976 | C_9_H_16_O_4_ | [M-H]- | 187, 125, 97 | 1.89 | 4.00×10^−10^ | 0.37 |
| Fulgidic Acid | 15.31 | 327.2175 | C_18_H_32_O_5_ | [M-H]- | 327, 229, 171 | 7.57 | 3.00×10^−9^ | 0.34 |
| Pinellic Acid | 15.88 | 329.2334 | C_18_H_34_O_5_ | [M-H]- | 329, 229, 211, 171 | 4.79 | 4.00×10^−9^ | 0.37 |
| Dimorphecolic Acid | 20.92 | 295.2277 | C_18_H_32_O_3_ | [M-H]- | 277, 195, 171 | 5.40 | 1.00×10^−7^ | 0.45 |
| **Other compounds** |  |  |  |  |  |  |  |  |
| Guanosine | 3.70 | 282.0843 | C_10_H_13_N_5_O_5_ | [M-H]- | 150, 133, 108 | 4.63 | 8.00×10^−12^ | 5.60 |
| Shogaol | 20.05 | 277.2159 | C_17_H_24_O_3_ | [M+H]+ | 135, 93, 91, 79 | 7.99 | 1.00×10^−7^ | 0.36 |

**Figure S1**

**
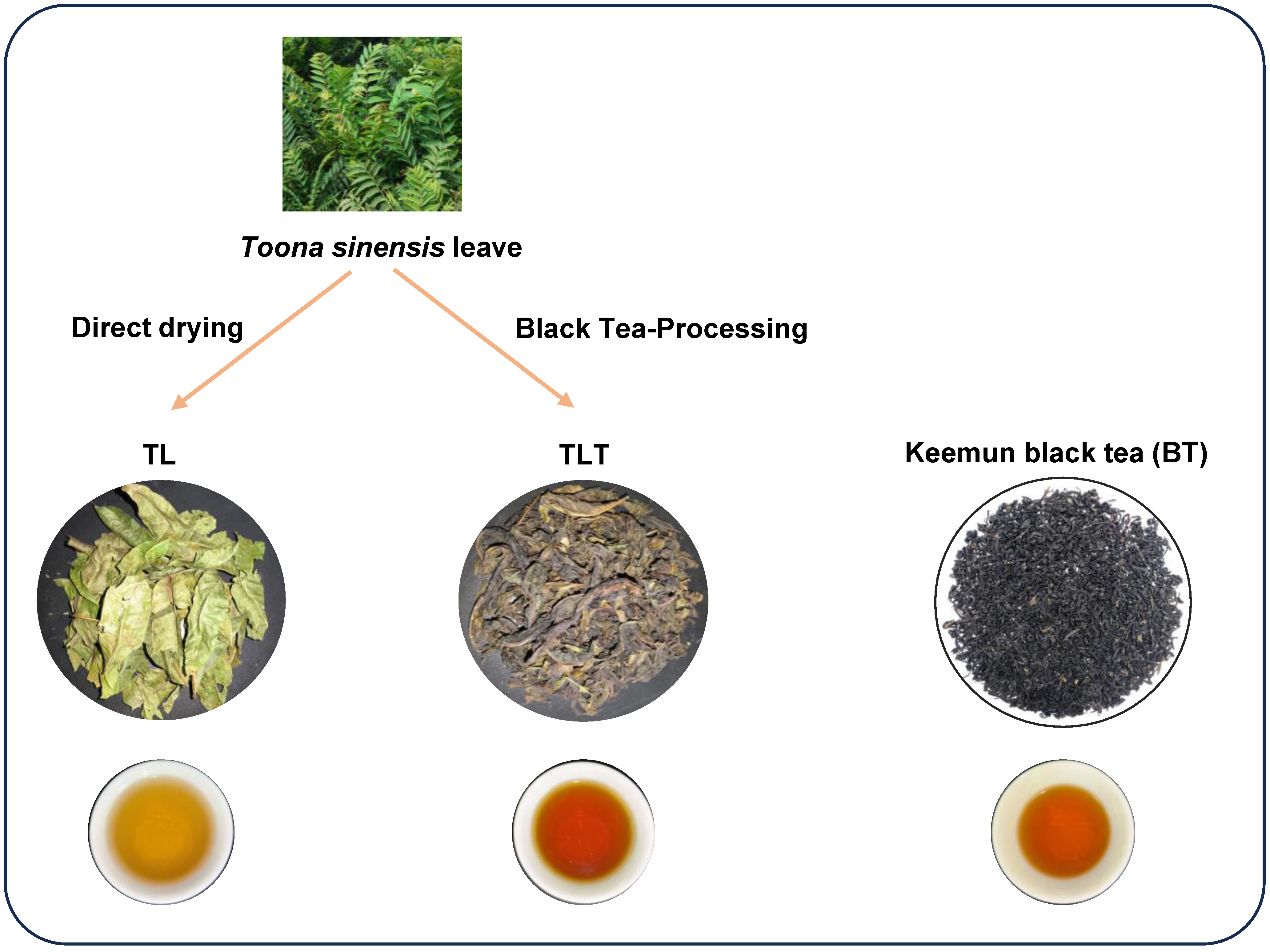
**

Figure S1. Appearance of fresh *Toona sinensis* leaves, hot air-dried leaves (TL), and black tea-processed *Toona sinensis* leaf tea (TLT).

**Figure S2**

**
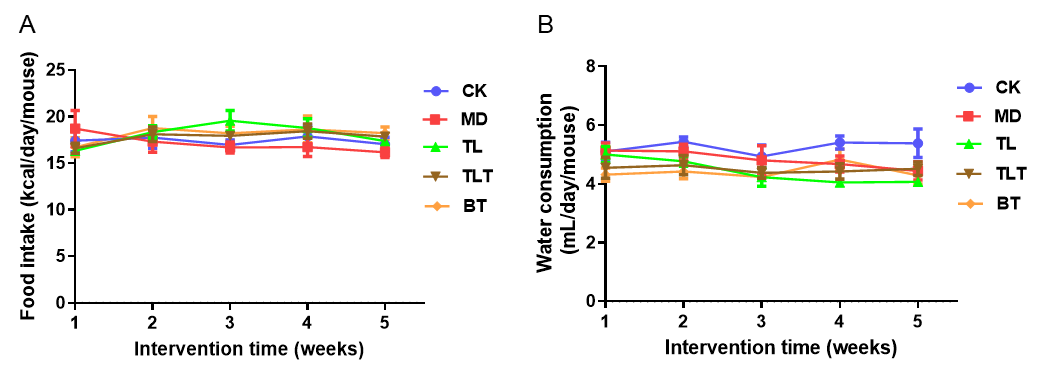
**

Figure S2. Food intake and water consumption in mice of different groups.

**Figure S3**

**
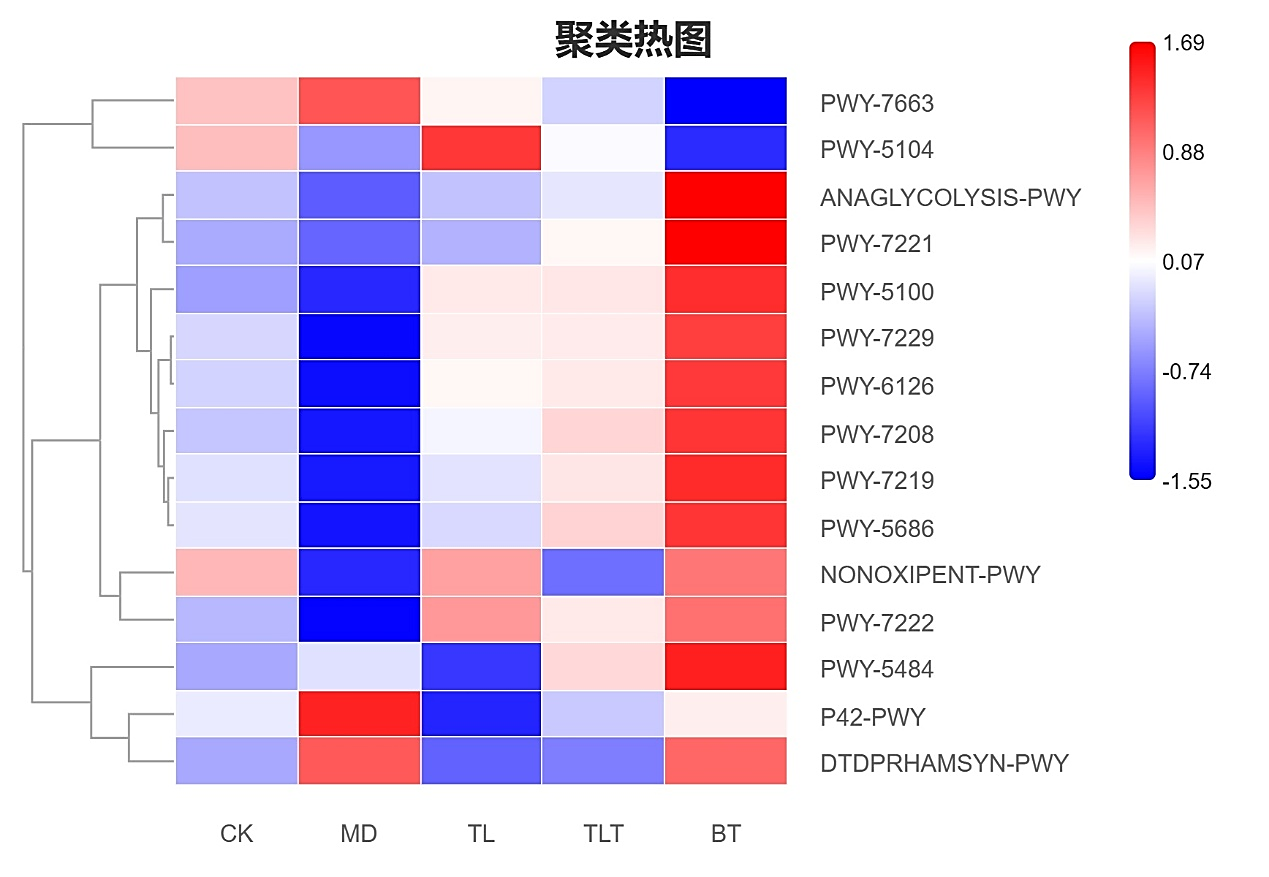
**

Figure S3. PICRUSt2-predicted MetaCyc pathways related to gut health. Heatmap showing Z-scored abundance of pathways across groups. TLT treatment reversed DSS-induced suppression of nucleotide biosynthesis and SCFA-related pathways. Red and blue indicate higher and lower pathway abundance, respectively.
